# Opening the Door for the New Era of Managing Sepsis-induced Coagulopathy

**Published:** 2026-09-07

**Authors:** KENTA KONDO, MAKOTO HIKI, TOSHIAKI IBA, YUTAKA KONDO

**Affiliations:** 1 Department of Emergency and Disaster Medicine, Juntendo University Graduate School of Medicine, Tokyo, Japan; 1 Department of Emergency and Disaster Medicine, Juntendo University Graduate School of Medicine, Tokyo, Japan; 2 >Faculty of Medical Science, Juntendo University, Chiba, Japan; 2 Faculty of Medical Science, Juntendo University, Chiba, Japan

**Keywords:** sepsis, disseminated intravascular coagulation, anticoagulant, inflammation, thrombolysis

## Abstract

Sepsis-associated disseminated intravascular coagulation (DIC) is a dynamic thromboinflammatory syndrome characterized by dysregulated coagulation, impaired fibrinolysis, platelet and leukocyte activation, endothelial injury, and microvascular thrombosis, ultimately leading to organ dysfunction and increased mortality. Recent advances in the understanding of its pathophysiology have shifted the concept of DIC from an irreversible end-stage complication to a potentially reversible condition when recognized and treated early. The updated 2025 International Society on Thrombosis and Haemostasis (ISTH) definition emphasizes disease progression from an early, often asymptomatic phase to overt DIC, supporting stage-specific diagnosis and intervention. In particular, recognition of sepsis-induced coagulopathy (SIC) as a precursor to overt DIC provides a valuable therapeutic window before irreversible organ damage develops. Current therapeutic strategies include heparins, physiological anticoagulants, and recombinant thrombomodulin, although their clinical benefits remain inconsistent because of patient heterogeneity and bleeding complications. Emerging approaches, including factor XI inhibitors, neutrophil elastase inhibitors, targeted fibrinolytic therapies, and nanotechnology-based drug delivery systems, aim to preserve antithrombotic efficacy while minimizing bleeding risk. Future management of sepsis-associated DIC will depend on earlier diagnosis, accurate disease staging, risk stratification, and personalized therapeutic strategies tailored to distinct coagulation phenotypes, thereby opening a new era in improving outcomes for patients with septic coagulopathy.

## Thromboinflammation in sepsis-associated DIC

The pathophysiology of sepsis-associated disseminated intravascular coagulation (DIC) is highly intricate, involving multiple overlapping mechanisms that disrupt hemostatic balance^1)^. While activation of the coagulation cascade is a hallmark feature, additional factors such as impaired fibrinolysis, excessive platelet and leukocyte activation, damage- associated molecular patterns (DAMPs), and neutrophil extracellular traps (NETs) release, and widespread endothelial dysfunction contribute significantly to disease progression. Fibrinolysis is commonly suppressed following the temporal activation due to elevated levels of plasminogen activator inhibitor-1 (PAI-1) and thrombin-activatable fibrinolysis inhibitor (TAFI), reducing clot breakdown^2)^. Platelets and leukocytes, beyond their fundamental roles, exhibit proinflammatory and procoagulant functions. Activated platelets express P-selectin and toll-like receptors (TLRs), enhancing leukocyte recruitment and complement activation^3)^. Simultaneously, endothelial cells lose their protective glycocalyx layer in response to systemic inflammation, exposing tissue factor (TF) and von Willebrand factor, which drive thrombin generation and promote platelet aggregation. This culminates in a hypercoagulable state, marked by microvascular thrombosis and impaired organ perfusion^4)^(Figure 1). In parallel, natural anticoagulant mechanisms—such as antithrombin and protein C—are diminished, further tipping the balance toward thrombosis^5)^. These interlinked processes define the concept of thromboinflammation, where immune, coagulation, and endothelial dysfunctions synergistically lead to multi-organ failure and increased mortality^6)^. Understanding these mechanisms is essential for developing targeted therapies aimed at modulating coagulation and inflammation in sepsis.

**Figure 1:**
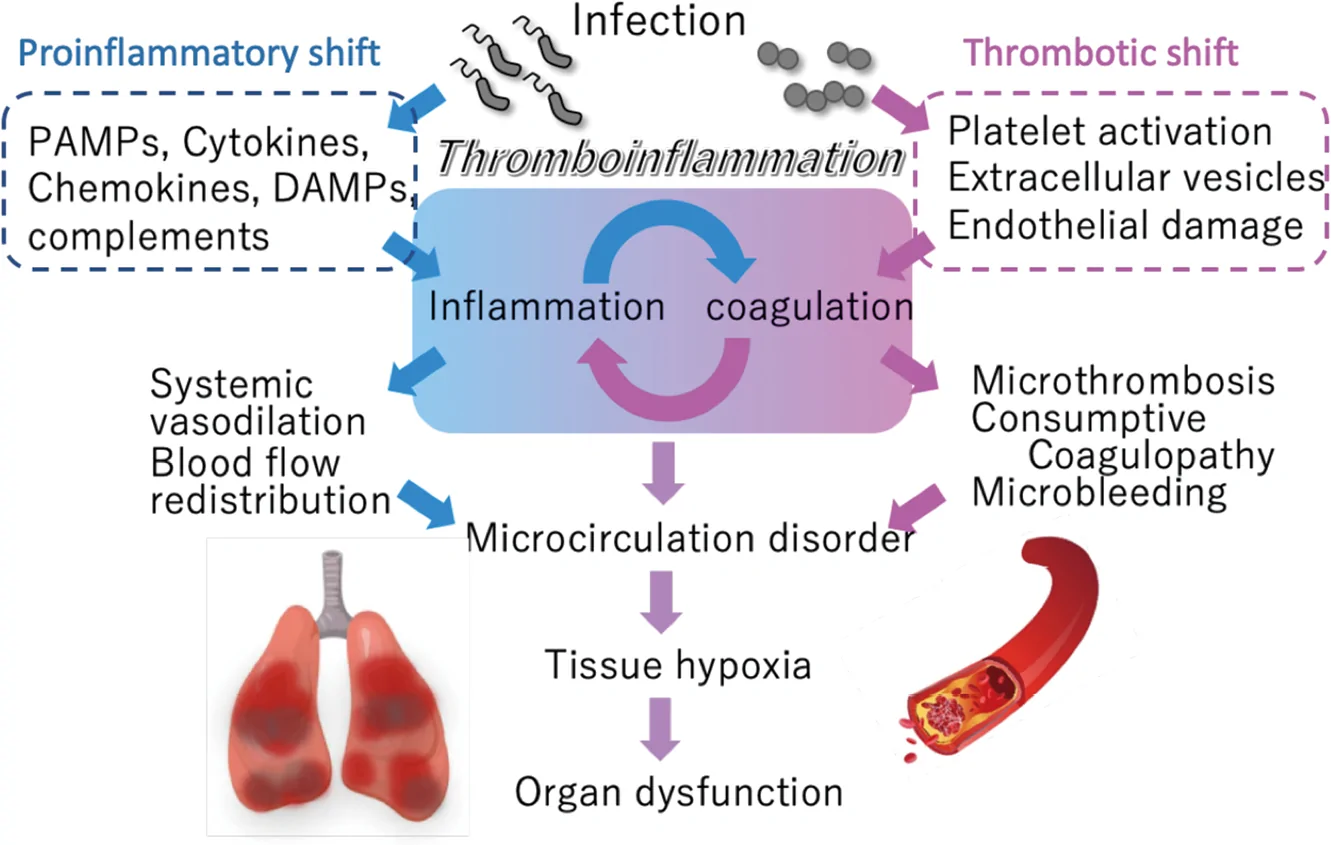
Pathomechanism of sepsis-induced organ dysfunction In sepsis, both inflammatory and coagulation pathways are activated and closely interact with one another (Thromboinflammation). The progression of inflammation is driven by multiple mediators, including cytokines, chemokines, pathogen-associated molecular patterns (PAMPs), damage-associated molecular patterns (DAMPs), and the complement system. In addition to coagulation activation, platelet activation, extracellular vesicles, and endothelial injury also contribute to thrombotic changes. These processes collectively result in microcirculatory disturbances, tissue hypoxia, and ultimately, organ dysfunction.

## A paradigm shift in DIC diagnosis

DIC has traditionally been regarded as a critical and largely untreatable complication of various underlying diseases, including sepsis, trauma, and malignancy. It was commonly viewed as a decompensated end-stage process in which therapeutic interventions had minimal impact, and clinical management was often limited to supportive care^7)^. However, this perception is shifting. Recognizing that early identification and intervention may alter the clinical course of DIC, the International Society on Thrombosis and Haemostasis (ISTH) Scientific and Standardization Committee released an updated definition and diagnostic criteria for DIC in 2025^8)^. This new framework redefines DIC as “an acquired, life-threatening intravascular disorder characterized by systemic coagulation activation, dysregulated fibrinolysis, and endothelial injury, resulting in microthrombosis. DIC arises from various underlying etiologies and progresses from a potentially asymptomatic early phase to an advanced phase with hemorrhage and/or organ dysfunction.” Importantly, the ISTH now acknowledges that DIC evolves progressively—from a preclinical or early phase with subtle biomarker changes to an overt stage associated with severe symptoms. Furthermore, the pathophysiology of the early phase is considerably different based on the underlying diseases. Based on this understanding, the ISTH proposes tailored diagnostic criteria for both early and overt phases of DIC. The recognition of sepsis-induced coagulopathy (SIC) as a precursor to overt DIC has provided a valuable therapeutic window for early intervention. Recently, a simplified SIC-2 score based solely on platelet count and prothrombin time has been proposed to facilitate earlier and more practical identification of sepsis-associated coagulopathy without relying on organ dysfunction assessment. Such simplified approaches may enhance timely patient selection for future anticoagulant strategies^9, 10)^(Figure 2). This shift in definition underscores the growing consensus that DIC is no longer universally “untreatable,” but rather a dynamic and potentially reversible condition if detected and addressed in its early stages.

**Figure 2:**
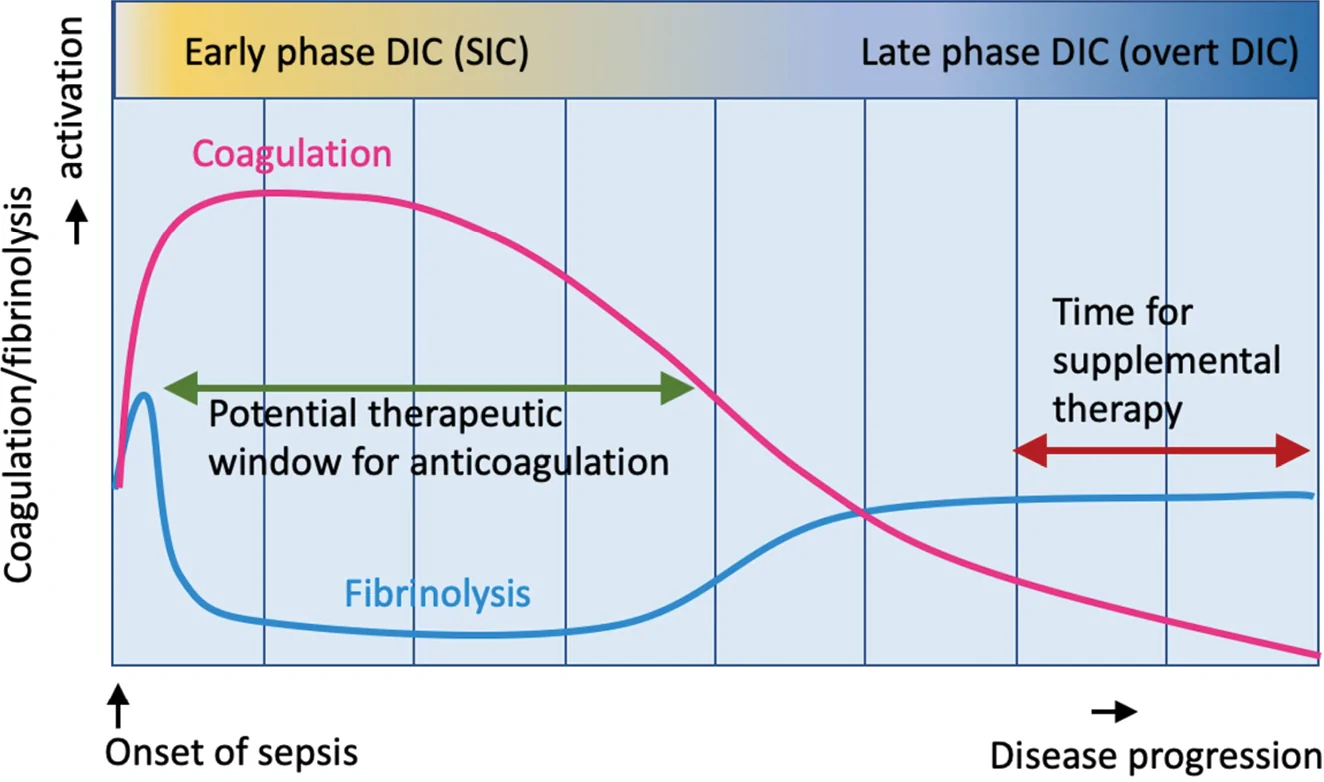
Changes in coagulation and fibrinolytic activity in sepsis The coagulation system is immediately activated after the onset of sepsis. The fibrinolytic system is also transiently activated by the release of tissue plasminogen activator (tPA). This temporal activation is suppressed continuously by the increased production of plasminogen activator inhibitor-1 (PAI-1). The therapeutic window for the anticoagulant therapy is the hypercoagulable early phase DIC.

## Future therapeutic opportunities

Treatment of DIC associated with sepsis targets the dysregulated coagulation and impaired fibrinolysis that contribute to microvascular thrombosis and organ failure. In this context, heparins are preferred for their long history and worldwide penetration. It may be beneficial, particularly in patients with persistent coagulopathy, though the reported effects on septic DIC are not consistent, and further study is necessary^11)^(Table 1). Besides, bleeding complications are highly risky, and they must be carefully assessed. On the other hand, physiological anticoagulants, such as protein C and antithrombin, have been known to have dual antithrombotic and anti-inflammatory properties, and were expected to be beneficial for treating severe sepsis. However, the results of the phase 3 trials were disappointing, and further studies examining the effects of these anticoagulants on septic DIC have not been done^12)^. Recombinant thrombomodulin is another anticoagulant that is currently used for DIC in Japan. It has shown potential in modulating both coagulation and inflammation in sepsis. However, its global acceptance remains limited due to inconclusive results in large international trials, emphasizing the need for further studies to identify responsive patient subgroups^13)^. It is noteworthy that although all the clinical studies using anticoagulants tended to show a beneficial effect, bleeding side effects cancel out their efficacy. Therefore, it will be difficult to succeed in clinical trials unless this obstacle is overcome. Beyond conventional anticoagulant therapies, a neutrophil elastase inhibitor (sivelestat) is expected to prevent tissue damage from activated neutrophils, especially to endothelial cells, and mitigate the progression of septic DIC^14)^. However, a prospective randomized trial is still lacking. Factor XI (FXI) inhibitors are emerging anticoagulant agents designed to prevent thrombosis by targeting the intrinsic coagulation pathway. Their unique mechanism offers the potential to reduce clot formation with a lower risk of bleeding compared to traditional anticoagulants. Animal models have demonstrated that FXI deficiency or inhibition can improve survival and reduce coagulopathy in sepsis-induced DIC scenarios^15)^. While FXI inhibitors have shown promise in reducing thrombotic events with minimal bleeding, there is currently a lack of clinical trials specifically evaluating their efficacy in DIC. Other than anticoagulant therapies, fibrinolytic therapy can be expected, especially for patients with a fibrinolysis-suppressive type of DIC. The nanogel spheres loaded with tPA are under development^16)^. There are some other advancements that represent promising directions in DIC therapy, focusing on targeted interventions that address both coagulation abnormalities and inflammatory processes. While some of them are still under investigation, they highlight the ongoing efforts to improve outcomes for patients with sepsis- associated DIC. Overall, managing sepsis-induced DIC requires early diagnosis, accurate staging, and risk stratification. It is also emphasized that tailored approaches based on DIC subtypes are mandatory. The modern view of DIC is shifting from an untreatable end-stage complication to a potentially reversible process, especially if intervention begins in the early phase of DIC.

**Table 1:** Summary of the anticoagulant/fibrinolytic therapies for DIC

Therapy	Mechanism/Target	Evidence/Notes
Low-dose heparin (UFH/LMWH)	Increase antithrombin activity and increase anticoagulant effect	Early use improves survival in SIC (observational study); recommended for low bleeding risk patients (ref. 11)
Recombinant thrombomodulin (rhTM)	Enhances protein C activity and modulates coagulation and inflammation	SCARLET trial: effective in subgroups with coagulopathy; widely used in Japan (ref. 13)
Antithrombin (AT)	Thrombin inhibition restores anticoagulant and antiinflammatory activity	KyberSept: no survival overall; potential role in selected DIC subgroups
Neutrophil elastase inhibitor (Sivelestat)	NET suppression; preserves fibrinolysis	Benefits in ARDS with DIC phenotype; more studies needed (ref. 14)
FXI inhibitors (e.g., ASO, monoclonals)	Inhibits the intrinsic pathway; minimal bleeding risk	Promising thrombosis prevention; no septic DIC-specific trials have been done (ref. 15)
Nanogel spheres loaded with tPA	Help fibrin dissolution and improve tissue circulation	The study for DIC is yet to be done, but the preclinical study is promising (ref. 16)
Crizanlizumab	A monoclonal antibody to P-selectin, involved in leukocyte adhesion and platelet aggregation	Approved for reducing the frequency of vaso-occlusive crises in sickle cell disease. No clinical trials have been done in DIC.
Nano-robotic thrombolysis	Navigate bloodstream to deliver thrombolytic agents directly to clots	No clinical evidence, but preclinical studies explored the potential efficacy

DIC: disseminated intravascular coagulation, UFH: unfractionated heparin, LMWH: low molecular weight heparin, SIC: sepsis-induced coagulopathy, NET: neutrophil extracellular trap, ARDS: acute respiratory distress syndrome, FXI: factor XI, ASO: antisense oligonucleotide, tPA: tissue plasminogen activator

## Conclusion

Sepsis-associated DIC should no longer be viewed as an inevitable terminal complication but as a dynamic and potentially modifiable thromboinflammatory syndrome. Advances in the understanding of coagulation, endothelial biology, and innate immunity have created new opportunities for earlier diagnosis and more precise intervention. The updated ISTH definition, together with simplified diagnostic approaches such as SIC-2, may facilitate identification of patients during the therapeutic window when anticoagulant strategies are most likely to be effective. This transition from treating overt DIC to identifying and targeting early thromboinflammation represents a fundamental paradigm shift in sepsis-associated DIC^17)^. The integration of earlier diagnosis, biomarker-guided patient selection, and safer precision therapeutics may ultimately transform the management of sepsis-associated DIC. By shifting the focus from overt DIC to the early thromboinflammatory phase, emerging diagnostic strategies and precision therapeutics may finally open the door to a new era in the management of sepsis-induced coagulopathy.

## Data availability

The data that support the findings of this study are available from the corresponding author upon reasonable request.

## Author contributions

KK and TI wrote the draft, and MH and YK significantly reviewed the manuscript. All authors read and approved the final manuscript.

## Conflicts of interest statement

The authors declare that they have no conflict of interest. TI is a member of the JMJ editorial board and was not involved in the peer review or decision-making process for this paper.
